# Determinants of Kidney Failure in Primary Hyperoxaluria Type 1: Findings of the European Hyperoxaluria Consortium

**DOI:** 10.1016/j.ekir.2023.07.025

**Published:** 2023-08-04

**Authors:** Elisabeth L. Metry, Sander F. Garrelfs, Lisa J. Deesker, Cecile Acquaviva, Viola D’Ambrosio, Justine Bacchetta, Bodo B. Beck, Pierre Cochat, Laure Collard, Julien Hogan, Pietro Manuel Ferraro, Casper F.M. Franssen, Jérôme Harambat, Sally-Anne Hulton, Graham W. Lipkin, Giorgia Mandrile, Cristina Martin-Higueras, Nilufar Mohebbi, Shabbir H. Moochhala, Thomas J. Neuhaus, Larisa Prikhodina, Eduardo Salido, Rezan Topaloglu, Michiel J.S. Oosterveld, Jaap W. Groothoff, Hessel Peters-Sengers

**Affiliations:** 1 Department of Pediatric Nephrology, Emma Children’s Hospital, Amsterdam UMC, University of Amsterdam, Amsterdam, The Netherlands; 2 Service de Biochimie et Biologie Moléculaire, UM Pathologies Héréditaires du Métabolisme et du Globule Rouge, Hospices Civils de Lyon, France; 3 Department of Nephrology, Catholic University of the Sacred Heart, Rome, Italy; 4 Centre de Référence des Maladies Rares Néphrogones, Hospices Civils de Lyon et Université Claude-Bernard Lyon 1, Lyon, France; 5 Institute of Human Genetics, Center for Molecular Medicine Cologne, University Hospital of Cologne, Cologne, Germany; 6 Center for Rare and Hereditary Kidney Disease Cologne, University Hospital of Cologne, Cologne, Germany; 7 Department of Pediatric Nephrology, Center Hospitalier Universitaire Liège, Liège, Belgium; 8 Department of Pediatric Nephrology, Assistance Publique–Hôpitaux de Paris Robert-Debré, University of Paris, Paris, France; 9 Department of Internal Medicine, University Medical Center Groningen, University of Groningen, Groningen, The Netherlands; 10 Department of Pediatrics, Pediatric Nephrology Unit, Bordeaux University Hospital, Bordeaux, France; 11 Department of Nephrology, Birmingham Women’s and Children’s Hospital NHS Foundation Trust, Birmingham, UK; 12 Department of Nephrology, University Hospitals Birmingham NHS Foundation Trust, Birmingham, UK; 13 Genetic Unit and Thalassemia Center, San Luigi University Hospital, Orbassano, Italy; 14 Institute of Biomedical Technology, CIBERER, University of Laguna, San Cristóbal de La Laguna, Spain; 15 Division of Nephrology, University Hospital Zurich, Zurich, Switzerland; 16 UCL Department of Renal Medicine, Royal Free Hospital, London, UK; 17 Department of Pediatrics, Children’s Hospital Lucerne, Lucerne, Switzerland; 18 Department of Inherited and Acquired Kidney Diseases, Veltishev Research and Clinical Institute for Pediatrics and Pediatric Surgery of the Pirogov Russian National Research Medical University, Moscow, Russia; 19 Department of Pathology, Center for Biomedical Research on Rare Diseases, Hospital Universitario Canarias, Universidad La Laguna, Tenerife, Spain; 20 Division of Pediatric Nephrology, Department of Pediatrics, Hacettepe University Faculty of Medicine, Ankara, Turkey; 21 Center for Experimental and Molecular Medicine, Amsterdam UMC, University of Amsterdam, Amsterdam, The Netherlands

**Keywords:** kidney failure, nephrocalcinosis, primary hyperoxaluria, urinary oxalate, urolithiasis, urinary glycolate

## Abstract

**Introduction:**

Primary hyperoxaluria type 1 (PH1) has a highly heterogeneous disease course. Apart from the c.508G>A (p.Gly170Arg) *AGXT* variant, which imparts a relatively favorable outcome, little is known about determinants of kidney failure. Identifying these is crucial for disease management, especially in this era of new therapies.

**Methods:**

In this retrospective study of 932 patients with PH1 included in the OxalEurope registry, we analyzed genotype-phenotype correlations as well as the impact of nephrocalcinosis, urolithiasis, and urinary oxalate and glycolate excretion on the development of kidney failure, using survival and mixed model analyses.

**Results:**

The risk of developing kidney failure was the highest for 175 vitamin-B6 unresponsive (“null”) homozygotes and lowest for 155 patients with c.508G>A and c.454T>A (p.Phe152Ile) variants, with a median age of onset of kidney failure of 7.8 and 31.8 years, respectively. Fifty patients with c.731T>C (p.Ile244Thr) homozygote variants had better kidney survival than null homozygotes (*P* = 0.003). Poor outcomes were found in patients with other potentially vitamin B6-responsive variants. Nephrocalcinosis increased the risk of kidney failure significantly (hazard ratio [HR] 3.17 [2.03–4.94], *P* < 0.001). Urinary oxalate and glycolate measurements were available in 620 and 579 twenty-four-hour urine collections from 117 and 87 patients, respectively. Urinary oxalate excretion, unlike glycolate, was higher in patients who subsequently developed kidney failure (*P* = 0.034). However, the 41% intraindividual variation of urinary oxalate resulted in wide confidence intervals.

**Conclusion:**

In conclusion, homozygosity for *AGXT* null variants and nephrocalcinosis were the strongest determinants for kidney failure in PH1.

## Graphical abstract

**Figure:**
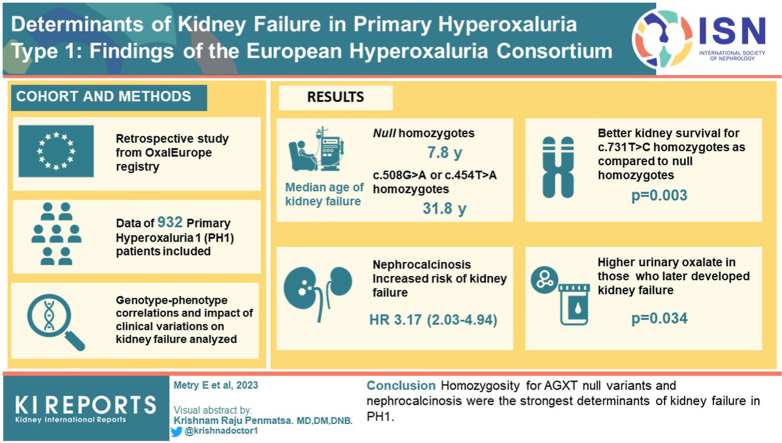

PH1 is a genetic metabolic disease, with an estimated prevalence of 1:152 000 in Europe and North America,^1^ whereas it is more common in the Middle East.^2^^,^^3^ International collaboration has enabled the establishment of the OxalEurope registry containing data from over 900 patients with PH1. PH1 is caused by the deficiency of alanine:glyoxylate aminotransferase (AGT) and results in hepatic overproduction of oxalate and glycolate. It is characterized by a highly heterogeneous disease course, ranging from the onset of kidney failure in the first year of life to occasional kidney stones in adulthood. *AGXT* genotype, nephrocalcinosis and urinary oxalate are factors assumed to determine kidney outcome. Nevertheless, few studies have investigated these associations and their findings require confirmation in larger patient groups.

More than 200 pathological *AGXT* variants are known to cause PH1.^4^ The c.508G>A (p.Gly170Arg) and c.454T>A (p.Phe152Ile) variants result in mistargeting of AGT to the hepatocytes’ mitochondrion instead of to the peroxisome, often combined with a reduced catalytic AGT activity. Patients with these missense variants may respond to treatment with vitamin B6, a precursor to pyridoxal 5’ phosphate, which is a cofactor for AGT. Vitamin B6 can partly correct the mitochondrial mistargeting and increases catalytic activity of AGT.^5^ Cohort studies confirm better kidney survival for patients with c.508G>A variants^6^ but c.454T>A (p.Phe152Ile) homozygotes have been less studied. Two other missense *AGXT* variants, c.731T>C (p.Ile244Thr) and c.121G>A (p.Gly41Arg), lead to protein aggregation and accelerated degradation, combined with partial mitochondrial mistargeting.^7^ It remains unclear whether patients with these variants have better kidney survival than patients with null mutations, who are intrinsically unresponsive to vitamin B6.

Little is known about other potential determinants of kidney failure in PH1. Nephrocalcinosis has been associated with a higher risk of developing kidney failure^8^^,^^9^; however, the impact is not entirely clear. In addition, the relationship between urinary oxalate excretion and kidney outcome in PH1 has not been widely studied, although the reduction in urinary oxalate is accepted as primary outcome measure for clinical trials in patients with PH1 with preserved kidney function.^10^

In this study, we used the OxalEurope registry to describe genotype-phenotype correlations, including potentially vitamin-B6 responsive missense variants that were previously only described in very small numbers. In addition, we investigated if nephrocalcinosis and urolithiasis were related to kidney outcome. Finally, we explored the association between urinary oxalate and glycolate excretion and the development of kidney failure.

## Methods

### Study Population and Design

This study is a retrospective analysis of data from patients with PH1, included in the OxalEurope registry on August 1, 2022. OxalEurope is a network of specialists from across Europe and beyond, involved in the treatment and research of primary hyperoxaluria. Patients were identified after presentation to one of the 20 participating OxalEurope centers. Informed consent was obtained according to the country’s regulations. We extracted the following data from patients with PH1: clinical data at presentation (sex, age, country of residence and origin, *AGXT* genotype, symptoms), age at diagnosis, 24-hour urinary oxalate and glycolate excretion, and kidney outcome at follow-up. The STROBE checklist was used as a guideline for reporting (Supplementary Material).

### Definitions

For descriptive statistics and for comparing the risk of kidney failure between patients with different *AGXT* pathogenic variants, we classified patients as follows: (i) homozygous B6+, (ii) compound heterozygous B6+/missense, (iii) homozygous missense, (iv) compound heterozygous B6+/null, (v) compound heterozygous missense/null, and (vi) homozygous null. The *AGXT* variants c.508G>A (p.Gly170Arg) and c.454T>A (p.Phe152Ile) were referred to with the term “B6+”, based on their DNA sequence change (missense), pathophysiological consequence (mitochondrial mistargeting) and evidence of *in vivo* vitamin B6 response.^11^ Infantile oxalosis was defined as the onset of kidney failure in the first year of life. Urinary oxalate and glycolate excretion were determined in 24-hour urine collections and reported as ratio (mmol per mmol creatinine, with age-dependent reference values) or absolute excretion (upper reference limit 0.50 mmol/24 hr per 1.73 m^2^).^12^ The primary outcome in this study was kidney failure, defined as estimated glomerular filtration rate <15 ml/min per 1.73 m^2^ or treatment by dialysis. This study did not include patients on RNA-interference therapy.

### Statistical Analyses

Categorical variables were presented as percentages and between-group differences were assessed using the chi-square test. Continuous variables were expressed as median (range or interquartile range [IQR]) and between-group differences were evaluated using Mann-Whitney U test and Kruskal-Wallis test. Cumulative survival rates were calculated from “date of birth” to “date of kidney failure” or censored at last follow-up date. Survival analysis was performed with Kaplan-Meier curves and log-rank test. The event of interest was kidney failure. Factors associated with kidney outcome were estimated by Cox-regression analyses. To meet the proportional hazards assumption, patients with infantile oxalosis were excluded from Cox-regression analyses for genotype. Patients with kidney failure at time of diagnosis were excluded from Cox-regression analyses for nephrocalcinosis and urolithiasis. Urinary oxalate and glycolate excretions determined after onset of kidney failure or after transplantation were excluded too. HRs, *P*-values, and 95% CI were provided. Mixed model analysis was performed to investigate the longitudinal outcome trajectory of urinary oxalate and glycolate excretion between patients with and without kidney failure at last follow-up, excluding measurements after onset of kidney failure. The mixed model was fitted on logarithmically transformed data, taking group, age, and their interaction as fixed effects; and patient-specific intercept and slope of time as random effects. Outcomes were back transformed to original scale for graphical interpretation. The method of calculating intraindividual variation was adapted from Clifford-Mobley *et al.*^13^ For each patient, the intraindividual variation was expressed as a coefficient of variation (CVintra) and calculated by the equation CVintra = SD/mean. The overall median value of CVintra was reported. All tests were 2-sided with a significance level of *P* < 0.05, using IBM SPSS version statistics version 28 (Armonk, NY, USA) and RStudio version 3.6.1 (RStudio Inc., Boston, MA, USA). The following R packages were used: ggplot2, survival, linear mixed-effects models, regression modeling strategies.

## Results

We obtained data of 932 patients (504 males) with PH1. Patients from France, the United Kingdom, Germany, the Netherlands, and Italy accounted for 76.0% of the cohort (Supplementary Figure S1). The clinical characteristics of all included patients are presented in Table 1.

**Table 1:** Baseline characteristics of patients with PH1, included in the OxalEurope registry

Patient characteristic	Overall *n* (%)	Available *n* (%)
Total number of included patients	932	
Sex (%)		917 (98.4)
Male	504 (55.0)	
Country of residency (%)		893 (95.8)
France	194 (21.7)	
United Kingdom	183 (20.5)	
Germany	148 (16.6)	
The Netherlands	95 (10.6)	
Italy	88 (9.9)	
Spain	31 (3.5)	
Belgium	24 (2.7)	
Other	130 (14.6)	
Genotype groups (%)		760 (81.5)
B6+/B6+	155 (20.4)	
B6+/missense	56 (7.4)	
missense/ missense	195 (25.7)	
B6+/null	120 (15.8)	
missense/null	52 (6.8)	
null/null	175 (23.0)	
AGXT double mutants^a^	7 (0.9)	
Signs and symptoms at time of diagnosis (%)		868 (93.1)
History of urolithiasis	521 (69.7)	
Nephrocalcinosis	433 (62.6)	
Both urolithiasis and nephrocalcinosis	242 (32.5)	
Recurrent urinary tract infections	91 (30.3)	
Kidney failure	362 (47.2)	
Age at first symptoms, median [IQR], yrs	4.0 [0.7–10.5]	478 (51.3)
Age at diagnosis (median [IQR]), yrs	8.0 [2.3–23.3]	673 (72.2)
Diagnostic delay (median [IQR]), yrs	1.0 [0.1–7.4]	458 (49.1)
Diagnosis to last follow-up (median [IQR]), yrs	5.7 [1.6–12.5]	598 (64.2)
Kidney failure at time of last follow-up	473 (63.9)	740 (79.4)
Age at onset of kidney failure (median [IQR]), yrs	17.0 [4.0–33.6]	
Infantile onset of kidney failure (%)	99 (10.6)	
Age at last follow-up (median [IQR]), yrs	17.8 [9.6–35.2]	647 (69.4)
Death at last follow-up^b^	87 (11.8)	740 (79.4)

AGXT, alanine-glyoxylate aminotransferase; IQR, interquartile range.

a AGXT double mutant: 2 mutations in cis in one allele.

b Death was preceded by kidney failure in all but 3 cases. Fifty-two patients died after having undergone (liver and/or-) kidney transplantation, the remaining number was on dialysis treatment.

### Clinical Characteristics and Kidney Outcome

*AGXT* genotype was known for 760 patients (81.5%, Table 1). There were 155 patients with B6+ homozygous variants (20.4%). A total of 195 (25.7%) patients were homozygous for other missense variants, most frequently for c.731 T>C (*n* = 64). Allelic frequencies of all individual missense variants are given in Supplementary Table S2. Median (IQR) age at symptom onset was 4.0 (0.7–10.5) years. The main presenting features were urolithiasis (69.7%) and nephrocalcinosis (62.6%). The median age at diagnosis was 8.0 (IQR 2.3–23.3) years. The median time between first symptoms and establishment of the diagnosis was 1.0 year (IQR 0.1–7.4). The median diagnostic delay was longer for adults (6.2 years) than for children (0.8 year). The median time between diagnosis and last follow-up in the entire cohort was 5.7 year (IQR 1.6–12.5). A total of 91 patients (13.5%) went unnoticed for at least a decade prior to establishment of the diagnosis, 72 of them were diagnosed prior to 2010.

**Figure 1:**
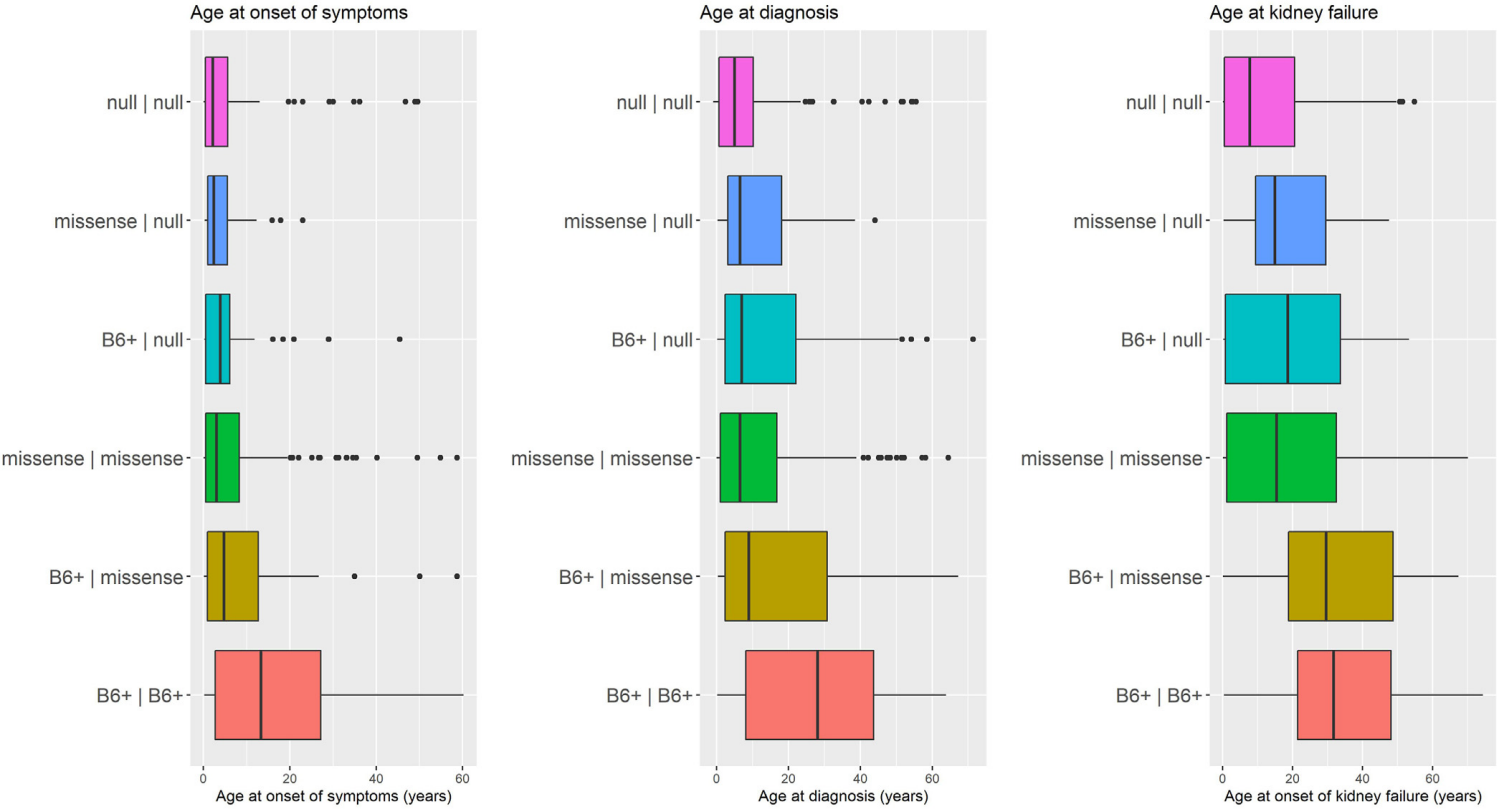
Age at onset of symptoms, age at diagnosis, and age at onset of kidney failure for patients with PH1 in genotype-based groups. Median and interquartile ranges for age at onset of symptoms, age at diagnosis, and age at onset of kidney failure. Dots represent outliers. PH1, primary hyperoxaluria type 1.

Kidney outcome was known for 740 patients. Clinical presentation of PH1 was related to *AGXT* genotype (Supplementary Table S1, Figure 1). Infantile oxalosis was very rare in B6+ homozygotes (*n* = 2, 1.6%), but occurred in a quarter of null homozygotes (*n* = 35, 25.0%). B6+ homozygotes were significantly older at time of first presentation, diagnosis, and onset of kidney failure (*P* < 0.001) as compared to other genotype groups. Nearly half of patients (47.2%) presented with kidney failure at the time of diagnosis. At last-follow-up, 473 patients (63.9%) had kidney failure.

A total of 198 patients with PH1 were diagnosed in adulthood. Seventy-three of them (41.0%) were B6+ homozygotes. A smaller number of patients were missense homozygotes (20.2%), B6+/null heterozygotes (14.6%), null homozygotes (9.0%), missense /null heterozygotes (7.3%), and B6+/missense heterozygotes (6.2%). They developed symptoms at a median age of 20.7 years and received the diagnosis at a median age of 37.7 years. At the time of diagnosis, the majority of adult patients with PH1 (71.5%) experienced kidney failure. The median age at onset of kidney failure was 35.4 years. Thirty patients died at a median age of 47.2 years. There were 362 (47.2%) patients whose diagnosis was missed until the onset of kidney failure. The percentages of patients in each genotype-based group were similar to those of the entire cohort. There was a large gap between median age at first symptoms (5.6 years) and median age at diagnosis (15.8 years). Patients who developed kidney failure had a median age of 15.3 years (IQR 0.9–34.1) at onset of kidney failure. This was not significantly different between patients who developed kidney failure prior to the establishment of the diagnosis or during follow-up (15.3 and 19.0 years, respectively). In 39 patients (4.2%), the diagnosis was only established after a failed kidney transplantation.

### Genotype-Phenotype Associations

**Figure 2:**
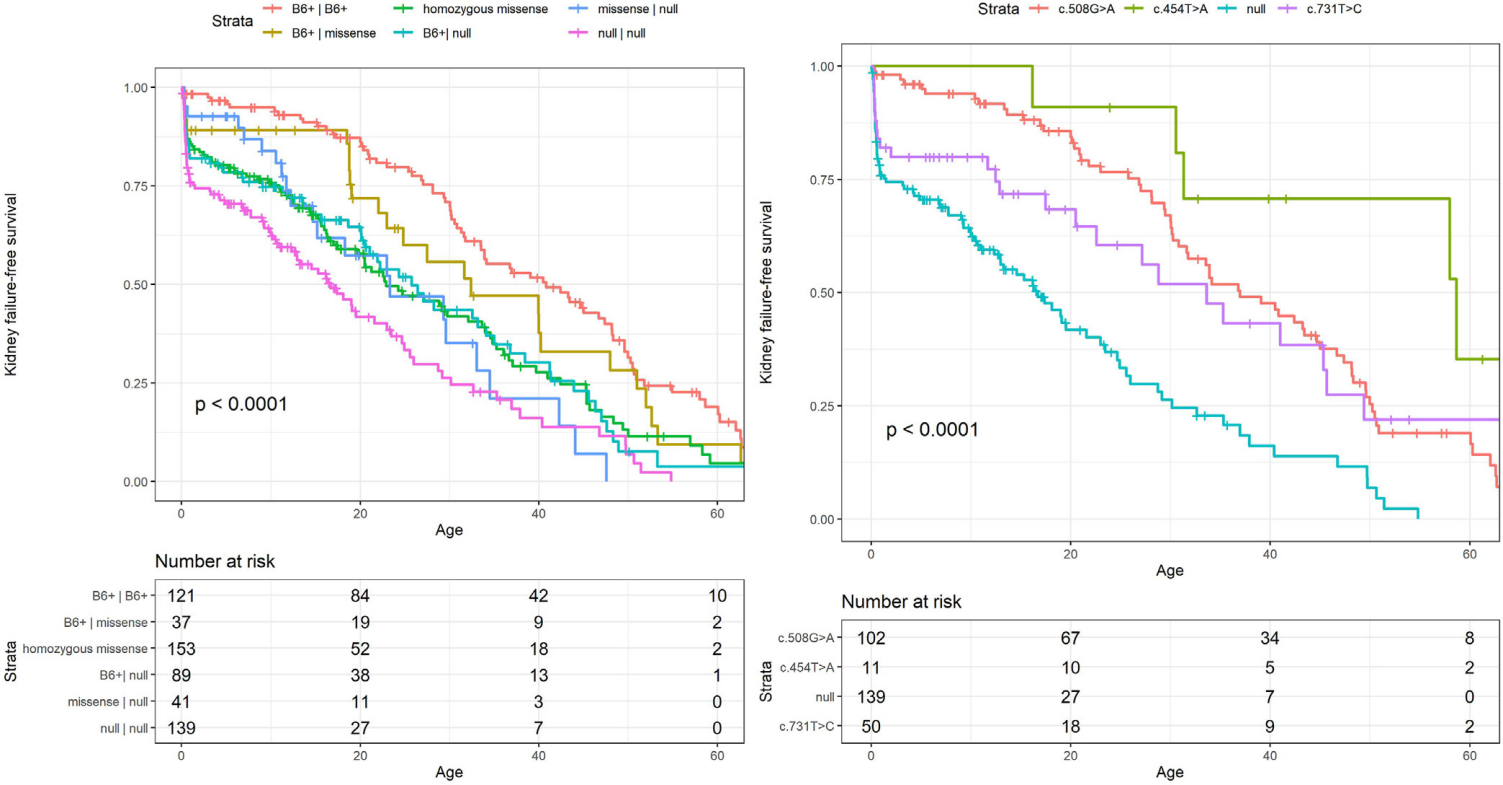
Kaplan-Meier survival curve for *AGXT* genotype-based groups of patients with PH1. The left survival curve displays kidney failure-free survival for the 6 genotype-based groups. The right survival curve displays kidney failure-free survival for patients with homozygous c.508G>A, c.731 T>C or null variants. PH1, primary hyperoxaluria type 1.

Compared to patients with B6+ homozygotes, all but 1 genotype-based group had a higher risk of developing kidney failure (Figure 2). This was most prominent in null homozygotes (HR 2.59; 95% CI [1.80–3.74], *P* < 0.001) and missense/null heterozygotes (HR 2.62; 95% CI [1.57–4.40], *P* < 0.001), but also the case for B6+/null heterozygotes (HR 1.59; 95% CI [0.63–1.07], *P* = 0.022) and missense homozygotes (HR 1.70; 95% CI [1.21–2.31], *P* = 0.002). In B6+/missense heterozygotes, the risk of kidney failure was not significantly higher than in B6+ homozygotes (HR 1.20; 95% CI [0.73–1.98], *P* = 0.464). Comparing null homozygotes to missense homozygotes, the risk of developing kidney failure was significantly higher in the first group (HR 1.46; 95% CI [1.00–2.11], *P* = 0.047). The median age at onset of kidney failure was 31.8 years for B6+ homozygotes, 29.6 years for B6+/missense heterozygotes, 15.4 years for missense homozygotes, 18.7 years for B6+/null heterozygotes, 15.0 years for missense/null heterozygotes and 7.8 years for null homozygotes (Figure 1). Comparing the 2 *AGXT* variants which were considered B6+, there was a trend of better kidney survival for patients with c.454T>A variants (*P* = 0.067, Figure 2).

### Individual AGXT Variants With Evidence for *In Vivo* Vitamin B6 Responsiveness

#### c.508G>A (p.Gly170Arg)

Kidney outcome was reported for 102 of 136 mostly Western European c.508G>A homozygotes. The median age at symptom onset in c.508G>A homozygotes was higher than that of the entire cohort (14.0; range 0.2–60.3) years. The median age at diagnosis was 28.1 years (range 0.2–63.4) years. There were 56 patients who presented with kidney failure. Twelve patients developed kidney failure during follow-up. The median age at onset of kidney failure was 31.6 (range 0.4–62.7) years. Fifteen patients died at a median age of 51.5 (range 0.7–67.6) years.

#### c.454 T>A (p.Phe152Ile)

Eleven patients were c.454T>A homozygotes, most of them from Western European countries. The median age at symptom onset was 19.6 (range 0.4–57.9) years and 30.9 years (range 8.4–59.3) at diagnosis. Ten patients had a history of urolithiasis at the time of diagnosis and nephrocalcinosis was found in 4 patients. In 2 patients, the diagnosis was only established after onset of kidney failure. Another patient had recurrent stone events from childhood on, but the diagnosis was only established in her 40s, and despite missing treatment with pyridoxine until then, kidney function had remained normal. There were no cases with infantile oxalosis. At the last follow-up, 6 patients had progressed to kidney failure at a median age of 44.7 (range 16.2–74.4) years. Three of them died at ages of 17, 45, and 62 years.

### Potential Candidate AGXT Variants for *In Vivo* Vitamin B6 Responsiveness

#### c.731 T>C (p.Ile244Thr)

Kidney outcome was known for 50 of 64 c.731T>C homozygotes (Figure 2). This variant was primarily found in patients from the Canary Islands and Northern Africa. The median age at symptom onset and diagnosis was 3.0 (range 0–49.6) and 8.0 (range 0–52.2) years, respectively. Nephrocalcinosis was found in 72.7% and urolithiasis in 68.8% of patients. As in the entire cohort, nearly half of patients (45.3%; *n* = 24) presented with kidney failure; 9 of them with infantile oxalosis. Four additional patients developed kidney failure during follow-up. The median age at onset of kidney failure was 12.9 (range 0.2–70.1) years. Six patients with kidney failure died at a median age of 25.5 (range 1.4–49.8) years. Kidney survival curves were not significantly different between c.731T>C and c.508G>A homozygotes but were borderline significant when comparing c.731T>C to c.454T>A homozygotes (*P* = 0.051). Comparing c.731T>C homozygotes to null homozygotes, kidney survival rates were significantly higher in c.731T>C homozygotes (*P* = 0.003).

Clinical characteristics and kidney outcome of patients with other *AGXT* missense variants, that are potential candidates for *in vivo* vitamin B6 responsiveness, are presented in Table 2. In these 34 patients, clinical presentation included family screening (*n* = 5), nephrocalcinosis (*n* = 4), and kidney stones (*n* = 11) to kidney failure (*n* = 14, including infantile oxalosis and failed kidney transplants). Vitamin B6 response was seen in 2 patients carrying c.121G>A variants and 1 patient heterozygous for c.139G>A. At last follow-up, 2 patients had developed kidney failure and 4 patients had died.

**Table 2:** Patient characteristics and kidney outcome of patients with PH1 with *AGXT* pathogenic missense variants, potentially responsive to vitamin B6

First allele	c.245G>A (p.Gly82Glu)	c.121G>A (p.Gly41Arg)			c.139G>A (p.Gly47Arg)		c.167T>A (p.Ile56Asn)		c.481G>A (p.Gly161Ser)	c.481G>T (p.Gly161Cys)
Second allele	c.245G>A (missense)	c.121G>A (missense)	c.508G>A (B6+)	c.466G>A (missense) c.847-1G>C (null) c.33dupC (*n* = 2, null)	c.508G>A(B6+)	c.847-1G>C (null)	c.167T>A (missense)	c.32C>G (missense)	c.33dupC (null)	c.614C>T (missense)
Number of patients	*n* = 8	*n* = 5	*n* = 7	*n* = 4	*n* = 1	*n* = 2	*n* = 1	*n* = 1	*n* = 3	*n* = 2
Country of origin	Pakistan (*n* = 4), other Asian countries (*n* = 4)	Turkey (*n* = 3), Italy (*n* = 1), Northern Africa (*n* = 1)	United Kingdom (*n* = 3), Russia (*n* = 2), France (*n* = 1), N.A. (*n* = 1)	United Kingdom (*n* = 1), France (*n* = 1), Germany (*n* = 1), N.A. (*n* = 1)	Italy	France	Tunisia	India	France (*n* = 2), Sweden (*n* = 1)	United Kingdom (*n* = 2)
Clinical presentation	Infantile oxalosis (*n* = 3), stones (*n* = 2), family screening (*n* = 1), kidney failure in childhood (*n* = 1), N.A. (*n* = 1)	Kidney failure (*n* = 1), failed kidney transplant (*n* = 1), stones (*n* = 2), family screening (*n* = 1)	Kidney failure and stones (*n* = 2), stones (*n* = 3), nephrocalcinosis (*n* = 1), N.A. (*n* = 1)	Kidney failure and stones (*n* = 2), nephrocalcinosis and stones (*n* = 2)	Failed kidney transplant	Failed kidney transplant (*n* = 1), family screening (*n* = 1)	Family screening in the second year of life	NC	Kidney failure (*n* = 1), nephrocalcinosis (*n* = 1), stones and nephrocalcinosis (*n* = 1)	Kidney failure (*n* = 1), family screening (*n* = 1)
Effect of vitamin B6 on urinary oxalate (Uox)	N.A.	Reduction (*n* = 1)	N.A.	Reduction (*n* = 1)	Reduction	N.A.	N.A.	N.A.	N.A.	Normal Uox even before B6 (*n* = 1)
Age at symptom onset, (median, range), yrs	0.3 (0.1–25.2)	N.A.	3.6 (1.3–6.1)	3.4, N.A. (*n* = 3)	35	23.1, asymptomatic (*n* = 1)	Asymptomatic	1.8	4, 12.3, N.A. (*n* = 1)	14.4 (*n* = 1), asymptomatic (*n* = 1)
Age at diagnosis (median, range), yrs	0.3 (0.1–25.5)	18.0 (2.8–33.8)	6.2 (1.4–54.3)	44.1 (17.8–50.1)	47.6	19.1, 23.9	1.9	2.0	4, 12.4, unknown (*n* = 1)	14.4, 9.8
Kidney outcome	Kidney failure (*n* = 4), no kidney failure (*n* = 3), N.A. (*n* = 1)	Kidney failure (*n* = 3), no kidney failure (*n* = 2)	Kidney failure (*n* = 2), no kidney failure (*n* = 3), N.A. (*n* = 2)	Kidney failure (*n* = 2), no kidney failure (*n* = 2)	Kidney failure	Kidney failure (*n* = 1), no kidney failure (*n* = 1)	Normal kidney function	N.A.	Kidney failure (*n* = 2), no kidney failure (*n* = 1)	Kidney failure (*n* = 1), no kidney failure (*n* = 1)
Age at onset of kidney failure, (median, range), yrs	0.3 (0.2–5.9)	29.2 (15.3–29.8)	32.4, 53.3	44.1 47.1	40.0	23.3	-	-	12.3, 15.2	14.4
Death at last follow-up	Three patients with kidney failure died	No deaths	No deaths	No deaths	Died	No deaths	No death	No death	No deaths	No deaths
Age at last follow-up (median, range), yrs	5.9 (2.0–25.9)	23.8 (14.8–42.8)	12.7 (3.4–55.1)	44.1 (27.8–49.3)	49.5	19.8 31.2	8.6	2.0	16.3 (11.8–16.6)	16.2, 20.4

N.A, not available; Uox, urinary oxalate.

### Nephrocalcinosis and Urolithiasis

**Figure 3:**
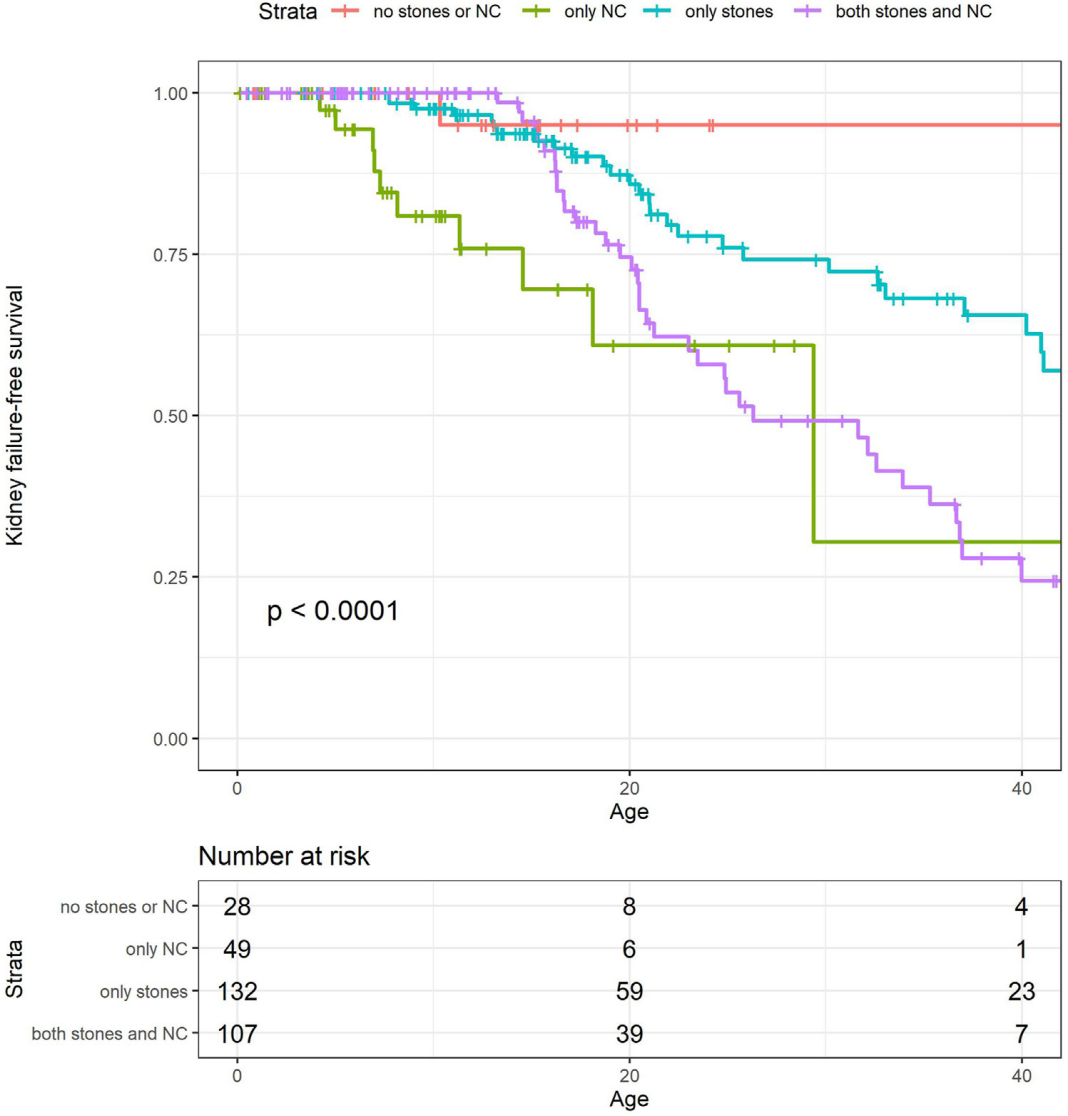
Kaplan-Meier survival curve for PH1 patient groups based on clinical features at time of diagnosis. Kidney-failure free survival rates for patients with no stones or NC (nephrocalcinosis), only NC, only stones, or both stones and NC. PH1, primary hyperoxaluria type 1.

Nephrocalcinosis increased the risk of developing kidney failure (HR 3.17 [2.03–4.94], *P* < 0.001) whereas urolithiasis did not (*P* = 0.870; Figure 3). In multivariate analysis, which included the age at diagnosis, the effect of nephrocalcinosis was still significant (HR 2.24 [1.43–3.50], *P* < 0.001).

### Urinary Biomarkers

**Figure 4:**
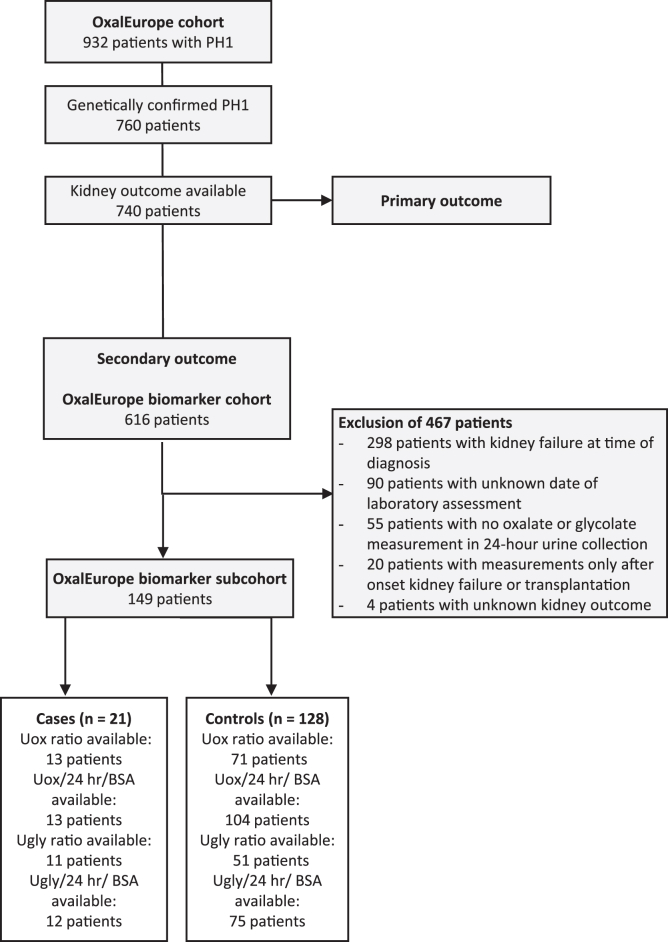
Flowchart. This flowchart shows the number of patients included in the OxalEurope registry (*N* = 932), the number of patients with genetically established PH1 (*n* = 760) and the number of patients with available data on kidney outcome at last follow-up (primary outcome; *n* = 740). In a subset of patients (*n* = 149), urinary oxalate and glycolate measurements were available and could be related to kidney outcome. The number of patients here correspond to the number of patients included in Figure 5 and 6. BSA, body surface area; Uox, urinary oxalate; Ugly, urinary glycolate.

As shown in the flowchart (Figure 4), urinary oxalate and glycolate measurements were available in 149 patients without kidney failure at baseline. Baseline characteristics were comparable for 24-hour urinary oxalate and glycolate excretion in those with and without kidney failure at follow-up (Supplementary Tables S3–S6).

### Oxalate

**Figure 5:**
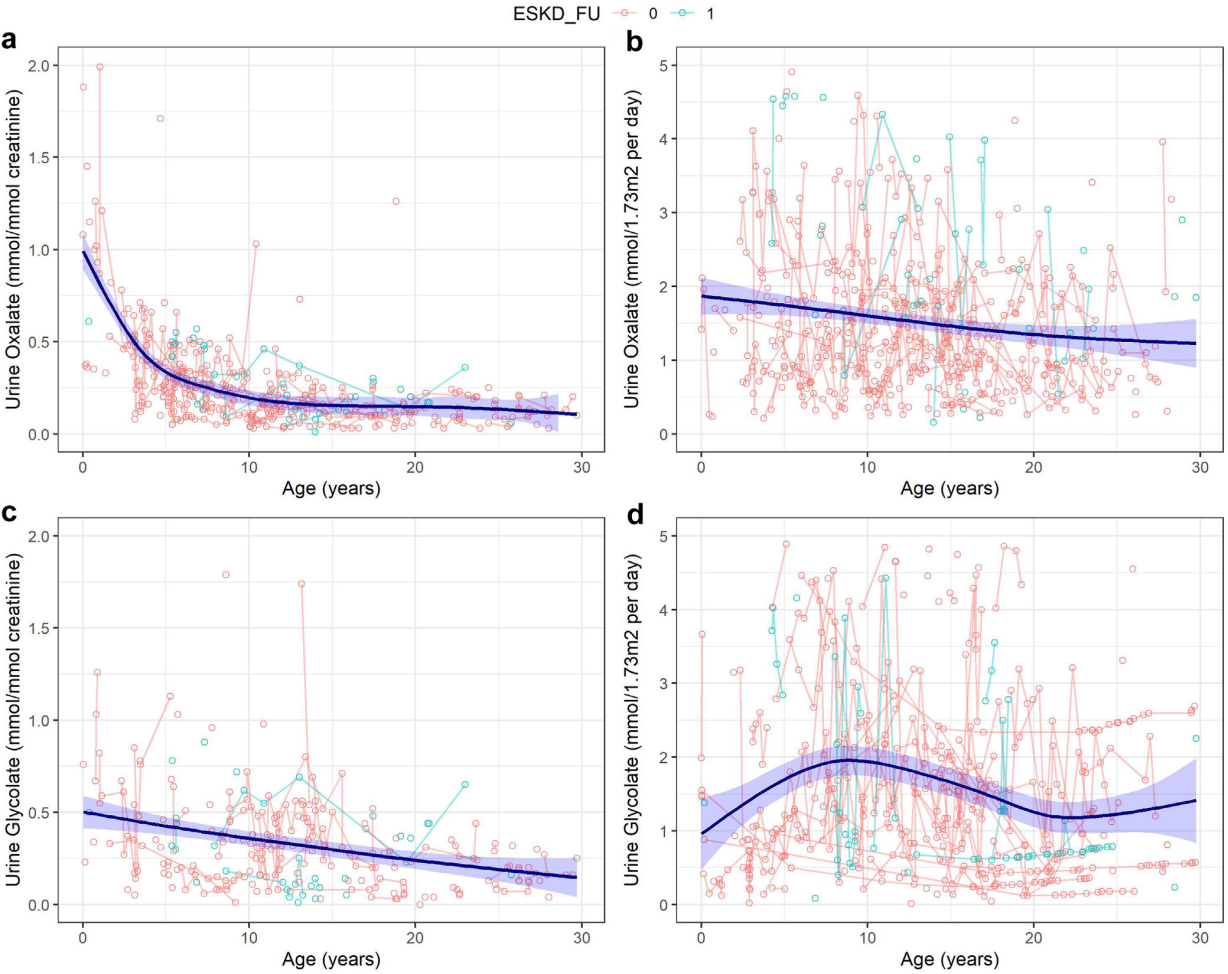
Urinary oxalate and glycolate excretion rates in patients with PH1 with and without kidney failure at last follow-up. All open circles represent measurements in 24-hour urine collections, measurements after onset of kidney failure or after transplantation were excluded. Green and red circles represent measurements in patients with and without kidney failure at last follow-up (ESKD, end-stage kidney failure = 1 and 0, respectively). Circles connected to each other represent repeated measurements in the same patient. Reference values of oxalate/creatinine and glycolate/creatinine ratios are respectively <0.17 and <0.29 mmol/mmol for children younger than 2 years, <0.10 and <0.23 mmol/mmol for children from 1 to 5 years, <0.08 and <0.19 mmol/mmol for children younger than 14 years, and <0.04 and <0.13 mmol/mmol for children older than 16 years. PH1, primary hyperoxaluria type 1.

**Figure 6:**
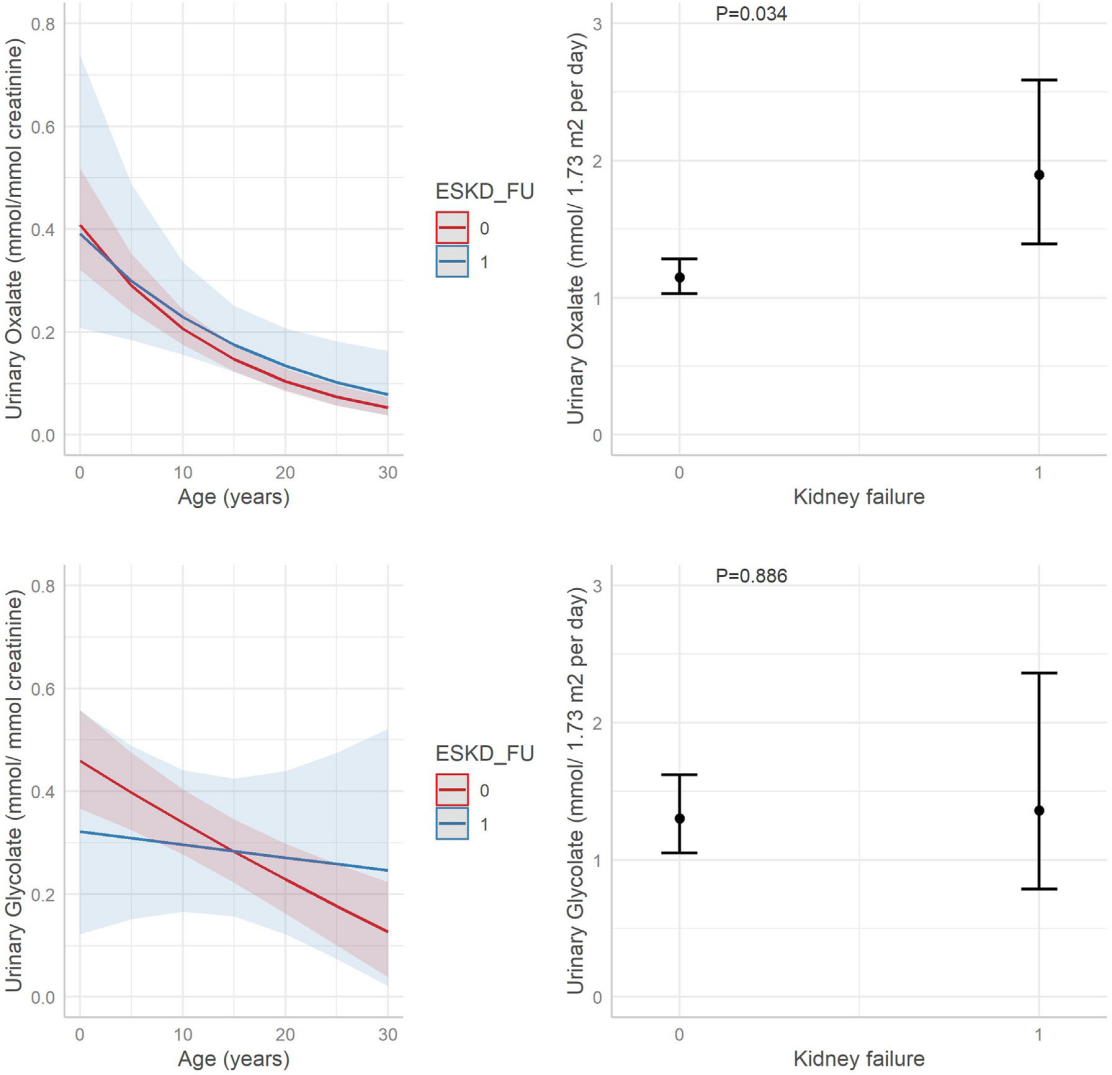
Mixed model analyses: urinary oxalate and glycolate excretion rates in patients with PH1 with and without kidney failure at last follow-up. Outcome trajectory of urinary oxalate and glycolate excretion between patients with and without kidney failure at last follow-up, excluding measurements after onset of kidney failure. The mixed model was fitted on logarithmically transformed data, taking group (with or without kidney failure at last follow-up), age (only for oxalate/creatinine and glycolate/creatinine ratios), and their interaction as fixed effects, and patient-specific intercept and slope of time as random effects. Outcomes were back transformed to original scale for graphical interpretation. PH1, primary hyperoxaluria type 1.

A total number of 470 urinary oxalate ratio measurements (oxalate/creatinine) were reported in 84 patients (Figure 5). The median (IQR) ratios were 0.99 (0.43–1.13) mmol/mmol creatinine for children younger than 1 year, 0.45 (0.26–0.55) mmol/mmol creatinine for children aged from 1 to 6 years, 0.22 (0.12–0.31) mmol/mmol for children aged from 6 to 12 years old, and 0.12 (0.06–0.18) for patients older than 12 years. Mixed model analysis did not reveal differences between urinary oxalate/creatinine ratios in patients with and without kidney failure (Figure 6). A total of 620 twenty-four-hour urinary collections were available from 117 patients. The median urinary oxalate excretion was 1.18 (IQR 0.81–1.80) mmol/24-hour per 1.73 m^2^. Thirty B6+ homozygotes had lower excretion rates than 18 cases with missense homozygous variants and 29 null homozygotes (medians 0.73, 1.40 and 1.74 mmol/24-hour per 1.73 m^2^, respectively, Supplementary Figure S2). Although there was a wide range of values in this group, urinary oxalate excretion was significantly higher in patients with kidney failure at the last follow-up (*P* = 0.034; Figure 6).

### Glycolate

There were 280 urinary glycolate ratio measurements available in 62 patients (Figure 5). The median (IQR) ratios were 0.50 (0.34–0.76) mmol/mmol creatinine for children younger than 1 year, 0.35 (0.23–0.55) mmol/mmol creatinine for children aged from 1 to 6 years, 0.36 (0.18–0.53) mmol/mmol creatinine for children aged from 6 to 12 years old, and 0.18 (0.08–0.34) mmol/mmol creatinine for patients older than 12 years. We found no difference in trajectories in patients with and without kidney failure (Figure 6). A total of 579 twenty-four-hour urinary glycolate excretion rates were available for 87 patients. Again, there were no differences in the trajectories (Figure 6). The median urinary glycolate excretion was 1.33 (IQR 0.77–2.87) mmol/24-hour per 1.73 m^2^. Twenty-three B6+ homozygotes had lower excretion rates than 12 missense homozygotes and 24 null homozygotes (medians 0.88, 0.88, and 2.74 mmol/24-hour per 1.73 m^2^, respectively, Supplementary Figure S3). There were 36 (58%) patients who had 1 or more (maximum 23) normal urinary glycolate excretion rates. In 30 of them, at least 1 sample revealed elevated urinary glycolate at some point. In the remaining 6 patients, there was only 1 measurement available. Thirteen patients were B6+ homozygotes, 12 patients were null homozygotes and 8 patients were compound heterozygotes (B6+/null). The remaining 3 patients were divided over the remaining 3 genotype-based groups.

### Intraindividual Variation

Median intraindividual variation was expressed as a coefficient of variation (CVintra = SD/mean). For urinary oxalate, CVintra was 43.1% for urinary oxalate ratios and 41.0% for 24-hour collections (mmol/24h per 1.73 m^2^). For urinary glycolate ratios, CVintra was 49.2% and for 24-hour urinary glycolate, CVintra was 57.4%.

## Discussion

Our study describes a unique cohort of 932 patients with PH1, evaluating their clinical and biochemical characteristics and their relation to kidney outcome. The unparalleled number of patients enabled us to investigate possible determinants of progression to kidney failure in PH1. Nearly half of the patient cohort presented with the outcome of interest, that is, kidney failure, which underlies the need for early diagnosis and treatment of PH1.

### Genotype-Phenotype Associations

In this study, we focused on genotype-phenotype correlations for *AGXT* variants with evidence for *in vivo* vitamin B6 responsiveness (c.508G>A and c.454T>A) and potential candidates (e.g. c.731T>C) according to a recent review that comprehensively summarized the current body of evidence for vitamin B6 responsiveness for several pathogenic variants.^11^ Genotype-phenotype associations were previously studied in 410 patients with PH1 registered in the OxalEurope database in December 2010.^6^ The current study confirms the previously described differences in the median age of onset of kidney failure in genotype-based patient groups (varying from 7.8 years in null homozygotes to >30 years for B6+ homozygotes or B6+/missense heterozygotes). Similarly, we found that the risk of developing kidney failure was 2.5 times higher in null homozygotes as compared to B6+ homozygotes. The actual differences between B6+ homozygotes and other genotype-based groups are even larger, because patients with infantile oxalosis were excluded from this analysis. The evidence for vitamin B6 responsiveness in patients with c.508G>A variants is relatively well established, both *in vitro* and *in vivo*.^5^^,^^6^ However, clinical outcomes of patients with other *AGXT* missense variants are scarce and are reported in detail in this study. Patients with c.454T>A variants may have better kidney survival than c.508G>A homozygotes, but this was not statistically significant in our cohort. This confirms previous findings in smaller cohorts.^14^ For the c.731T>C variant, the evidence supporting vitamin B6 responsiveness in the literature is conflicting. One study reported positive results *in vitro*;^15^ however, the only clinical evidence of vitamin B6-responsiveness was found in 1 compound heterozygous patient^16^ whereas a homozygous patient was found to be unresponsive.^17^ In this study, kidney survival of c.731T>C homozygotes was not significantly different from B6+ homozygotes, whereas the difference with null homozygotes was evident. Based on *in vitro* results, it has been suggested that pyridoxal and pyridoxamine could better counteract the protein misfolding, caused by the c.731T>C variant, than pyridoxine. In their cellular study, Oppici *et al.*^18^ found that these 2 active forms of vitamin B6 increased protein levels approximately 2.5-fold whereas pyridoxine had no effect. A clinical trial investigating the effect of different forms of vitamin B6 in this patient group would be an interesting next step. The c.245G>A variant was not related to a better kidney outcome. Although this variant is associated with preserved immune-reactive AGT, there is no catalytic activity *in vitro*^19^ and therefore likely no effect of vitamin B6. This finding is consistent with the poor clinical outcomes found in the 8 c.245G>A homozygotes. The c.121G>A variant leads to partial mistargeting of AGT, however pyridoxine had no effect *in vitro*,^19^ partly due to a reduced binding affinity for pyridoxal 5’ phosphate of the mutant protein. Only 1 case report has described a reduction in urinary oxalate of >50%, suggesting vitamin B6 responsiveness.^20^ In our cohort, 3 of 5 c.121G>A homozygotes developed kidney failure before the age of 30 years, though this did not preclude vitamin B6-responsiveness. According to *in vitro* studies, the c.139G>A, c.167T>A, c.481G>A, and c.481G>T variants potentially impart vitamin B6 responsiveness,^21^, ^22^, ^23^ however clinical outcomes were lacking so far. In every patient group (except for patients with the c.167T>A variant), there was at least 1 patient who presented with kidney failure in childhood or early adulthood. Taking into account that individual cases showed reduction of urinary oxalate after treatment with vitamin B6, these results stress the importance of early detection and testing of vitamin B6-responsiveness, which may be repeated, since an intra-individual difference in response over time is a possibility.^24^ It should be taken into account that patients with the same pathogenic variant may respond differently to vitamin B6.^24^ Finally, therapy adherence and intra-individual biological variation in urinary oxalate excretion could influence the measured response.

### Nephrocalcinosis and Urolithiasis

We found that nephrocalcinosis was associated with a three-fold higher risk of developing kidney failure, whereas urolithiasis was not. This is in line with an international cohort study of 170 patients with PH1,^8^ in which baseline characteristics were comparable to those of the current study cohort.

### Urinary Biomarkers

#### Oxalate

Oxalate/creatinine ratios were not significantly higher in patients who developed kidney failure, possibly related to the higher percentage of B6+ homozygotes in the group with favorable kidney outcome. Examining absolute urinary oxalate excretions, we did find significantly higher urinary oxalate in patients with kidney failure at last follow-up. However, the effect was small and the CI was wide. Only 1 previous study has investigated the impact of urinary oxalate on kidney outcome and found that urinary oxalate excretion at diagnosis, stratified by quartile, was strongly associated with the risk of developing kidney failure (HR 3.4 [1.4–7.9]).^25^ Unfortunately, this study described patients with several types of primary hyperoxaluria and the results for patients with PH1 were not presented separately. In addition, patients were stratified in quartile groups by baseline urinary oxalate excretion, not considering the high biological variability.

It is known that the variation in urinary oxalate excretion in healthy volunteers is high (42.5%).^26^ In this study, we found a CVintra of no less than 41.0%. Urinary oxalate excretion data in the OxalEurope registry reflect routine clinical practice (e.g., collection inaccuracies and treatment compliance), with measurements performed during clinical follow-up and not under standardized conditions. In addition, sample handling (e.g., urine acidification) and analysis methods may differ for each laboratory and may partly account for variation in urinary oxalate excretion caused by external factors. Intraindividual variation could also be attributed to varying oxalate absorption from the diet, intraluminal binding to calcium in the intestines, and processing in the gut. Clifford-Mobley *et al.*^13^ included 27 patients with PH1 who collected 24-hour urine samples under standardized conditions (including low-oxalate diet) and found a substantially lower median CVintra of 14% (range 0%–36%). The authors concluded that based on their data, a reduction in urine oxalate excretion of approximately one-third would be required to prove a clinically significant effect of any treatment under study. A higher CVintra, as found in our study, would require an even higher percentage reduction. Although urinary oxalate excretion is a main outcome in clinical trials with patients with PH1,^10^ it remains to be established what percentage reduction in urinary oxalate excretion will actually prevent progression to kidney failure. More insight into this will probably come from long-term follow-up data clinical trials with RNA-therapeutics, that effectively reduce urinary oxalate excretion.

#### Glycolate

An association between urinary glycolate excretion and kidney outcome was not found in this study. This biomarker, with a very high CVintra, was not necessarily elevated in every sample of patients with PH1, regardless of *AGXT* genotype. Considering that glycolate excretion is highly influenced by diet and that normal glycolate excretion rates do not exclude PH1, we recommend proceeding with genetic testing in case of high clinical suspicion of PH1 but normal urinary glycolate excretion, as proposed in the recent European guidelines.^27^

### Strengths and Limitations

The OxalEurope registry is the largest database available worldwide, comprising real-world observational data. The main strength of this study is the availability of data on genotype-phenotype correlations, with kidney failure as phenotypic outcome. The parallel weakness of this study is the lack of other phenotypic outcomes. Potential additional clinical outcomes include kidney function or chronic kidney disease stage, number of stone events, and grade of nephrocalcinosis. Including those outcomes would have given more information on the course of the disease in patients with different *AGXT* genotypes, whereas we now focus on the absence or presence of kidney failure at last follow-up. Besides genotype-phenotype correlations, there are several other factors assumed to influence the disease course, such as conservative treatment methods, which were not considered in our analyses. Even patients with unfavorable genotypes may thus experience a stable disease course if adequately treated, given that this (conservative or RNA-interference) treatment significantly influences outcome.

Several limitations are inevitable and inherent to the partly retrospective nature of the OxalEurope registry. Not all data were available in all patients. When analyses were performed in a subgroup of 932 patients for that reason, this was indicated in the manuscript or tables. Center-specific supply could have influenced the results if the provided care differs from center to center, for example with regard to monitoring adherence to vitamin B6 treatment. However, we think this effect is not significant because the main participating centers are located in Western Europe and would monitor their patients in the same way. Historically, OxalEurope did not consistently capture all variables of interest, such as the response and compliance to hyperhydration, urine alkalizers or vitamin B6 treatment. Vitamin B6 efficacy to reduce urinary oxalate excretion per patient was not routinely tested by standardized methods and only sporadically captured. Therefore, the term ‘B6+’ did not indicate actual biochemical vitamin B6 response; however, it was rather used to refer to patients with *AGXT* variants with evidence for *in vivo* vitamin B6 responsiveness (c.508G>A and c.454T>A).^11^ Better kidney survival rates in B6+ patients are not necessarily linked to the response of pharmacologic vitamin B6 but could also be the result of some amount of preserved AGT activity in patients with these 2 variants. Investigating the relationship of urinary biomarkers with the development of kidney failure, it appeared challenging to ‘catch’ the precise development of kidney failure in the disease course. Nearly half of patients had developed kidney failure prior to diagnosis and most others did not develop kidney failure during the limited follow-up duration. Although urine measurements in CKD 5 were excluded, the lack of data on kidney function implies that we could have included measurements in CKD 4, which may be falsely low due to impaired tubular secretion of oxalate in kidney failure.^28^ Finally, investigating the relationship between urinary oxalate and glycolate and kidney outcome can be seen as a strength, because this has not been done previously. At the same time, we recognize that these data were only available in a minority of patients. Unfortunately, this limited the interpretation of the effect of urinary oxalate on outcome. In addition, data were not collected under controlled circumstances, but rather reflected clinical practice.

### Conclusion

The main focus of this study was genotype-phenotype correlations; an unparalleled number of c.731T>C homozygotes was studied, revealing similar outcomes in this group as compared to c.508G>A or c.454T>A homozygotes. In addition, we found that nephrocalcinosis at time of diagnosis increased the risk of kidney failure 3 times. Urinary oxalate excretion was significantly higher in patients who developed kidney failure as compared to those with a favorable kidney outcome. Urinary glycolate was not associated with kidney outcome and repeated 24-hour urine collections were needed to reveal elevated excretion rates in some cases. Intraindividual variation was high for both biomarkers. A continued contribution of follow-up data will keep the OxalEurope registry updated, providing information on the prognosis of patients with PH1 in the era of new therapies.

## Disclosure

JWG, MJSO, SFG, LJD, and ELM have received an unconditional grant from both Alnylam Pharmaceuticals and Dicerna (Novo Nordisk) Pharmaceuticals to fund the OxalEurope Registry. SFG received a PhD scholarship from the Amsterdam University Medical Centers. SAH and SHM have received consultation fees from Alnylam Pharmaceuticals and Dicerna Pharmaceuticals. BBB, CA, GM, JB, JHa, JHo, LC, MJSO, and PMF have received consultation fees from Alnylam Pharmaceuticals. CMH has received consultation fees from Dicerna Pharmaceuticals and funding from Fundación Disa. All the other authors declared no competing interests.

## Supplementary Material

Supplementary File (PDF)

**Figure S1.** Absolute patient numbers included in the OxalEurope registry.

**Figure S2.** Urinary oxalate for B6+ homozygotes, missense homozygotes, and null homozygotes.

**Figure S3.** Urinary glycolate for B6+ homozygotes, missense homozygotes, and null homozygotes.

**Table S1.** Baseline characteristics of patients with PH1, groups based on *AGXT* genotype.

**Table S2.** Allelic frequency of missense variants in groups based on *AGXT* genotype.

**Table S3.** Baseline characteristics of patients with PH1 and available urinary oxalate ratio (mmol/mmol creatinine).

**Table S4.** Baseline characteristics of patients with PH1 and available 24-hour urinary oxalate excretion.

**Table S5.** Baseline characteristics of patients with PH1 with available urinary glycolate ratio.

**Table S6.** Baseline characteristics of patients with PH1 with available 24-hour urinary glycolate excretion.

**STROBE Checklist.**
