# Supplementary material for: Determinants of Kidney Failure in Primary Hyperoxaluria Type 1: Findings of the European Hyperoxaluria Consortium
Source: Kidney Int Rep. 2023 Aug 4;8(10):2029–42. doi: 10.1016/j.ekir.2023.07.025 (PMC10577369; doi:10.1016/j.ekir.2023.07.025)
Supplement: Supplementary File (PDF) [file mmc1.pdf]

Supplementary Material [.PDF]

Table of content

Table S1 Baseline characteristics of patients with PH1, groups based on *AGXT* genotype

Table S2 Allelic frequency of missense variants in groups based on *AGXT* genotype

Table S3 Baseline characteristics of PH1 patients and available urinary oxalate ratio (mmol/mmol creatinine)

Table S4 Baseline characteristics of PH1 patients and available 24-hour urinary oxalate excretion

Table S5 Baseline characteristics of PH1 patients with available urinary glycolate ratio

Table S6 Baseline characteristics of PH1 patients with available 24-hour urinary glycolate excretion

Figure S1 Absolute patient numbers included in the OxalEurope Registry

Figure S2 Urinary oxalate for B6+ homozygotes, missense homozygotes and null homozygotes

Figure S3 Urinary glycolate for B6+ homozygotes, missense homozygotes and null homozygotes

STROBE Checklist

Table S1 Baseline characteristics of PH1 patients, groups based on *AGXT* genotype

	B6+ | B6+	B6+ | missense	missense | missense	B6+ | null	missense | null	null | null	p-value
n	155	56	195	120	52	175	
Male sex (%)	78 (50.3)	27 (48.2)	109 (56.2)	75 (63.0)	28 (53.8)	98 (56.3)	0.332
Infantile oxalosis (%)	2 (1.6)	6 (14.3)	24 (15.6)	17 (18.3)	3 (7.0)	35 (25.0)	< 0.001
Age at first symptoms (median [IQR]), years	13.4 [2.7 – 27.2]	4.8 [0.9 – 12.8]	3.0 [0.6 – 8.3]	3.9 [0.5 – 6.1]	2.4 [1.0 – 5.5]	2.2 [0.4 – 5.6]	< 0.001
Age at diagnosis (median [IQR]) , years	28.1 [8.1 – 43.7]	9.0 [2.4 – 30.8]	6.6 [1.0 – 16.8]	7.0 [2.4 – 22.1]	6.6 [3.1 – 18.1]	5.0 [0.6 – 10.2]	< 0.001
Diagnostic delay (median [IQR])	3.8 [0.6 – 12.2]	1.0 [0.2 – 4.7]	0.9 [0.1 – 5.7]	0.9 [0.2 – 11.2]	1.0 [0.3 – 14.0]	0.4 [0.1 – 4.3]	0.002
Asymptomatic at time of diagnosis (%)	7 (6.7)	1 (3.3)	11 (8.3)	1 (1.3)	1 (2.8)	9 (8.1)	0.280
Clinical features at time of diagnosis (%)							0.004
- No nephrocalcinosis or urolithiasis	15 (10.9)	4 (9.5)	15 (9.3)	8 (7.3)	1 (2.3)	16 (11.4)	
- Only nephrocalcinosis	20 (14.6)	8 (19.0)	39 (24.2)	25 (22.7)	8 (18.2)	40 (28.6)	
- Only urolithiasis	63 (46.0)	18 (42.9)	51 (31.7)	56 (50.9)	15 (34.1)	38 (27.1)	
- Both nephrocalcinosis and urolithiasis	39 (28.5)	12 (28.6)	56 (34.8)	21 (19.1)	20 (45.5)	46 (32.9)	
Kidney failure at time of diagnosis (%)	62 (47.3)	17 (39.5)	80 (47.1)	48 (48.5)	14 (31.8)	74 (47.7)	0.429
Age at onset of kidney failure (median [IQR]), years	31.8 [21.4 – 48.2]	29.6 [18.8 – 48.7]	15.4 [1.1 – 32.5]	18.7 [0.8 – 33.6]	15.0 [9.4 – 29.5]	7.8 [0.5 – 20.6]	< 0.001
Age at last follow-up (median [IQR]), years	38.9 [20.7 – 54.7]	24.1 [13.9 – 46.4]	15.5 [8.8 – 29.9]	17.9 [10.6 – 35.8]	13.9 [8.5 – 26.4]	12.5 [5.4 – 20.8]	< 0.001

Table S2 Allelic frequency of missense variants in groups based on *AGXT* genotype

Pathogenic variant	Protein change	B6/missense group (n=56)	Missense/missense group (n=195)	Missense/null group (n=52)
c.2T>C	p.Met1Thr		1	
c.3G>T	p.Met1Ile	2		
c.22G>C	p.Val8Leu		2	
c.32C>G	p.Pro11Arg		1	
c.79G>T	p.Gly27Trp	1		
c.106C>T	p.Arg36Cys	3	4	
c.107G>A	p.Arg36His			
c.121G>A	p.Gly41Arg	7	11	4
c.122G>T	p.Gly41Val	1		1
c.122G>A	p.Gly41Glu	2		
c.139G>A	p.Gly47Arg	1		2
c.167T>A	p.Ile56Asn		3	
c.175G>A	p.Glu59Lys		2	
c.209C>A	p.Thr70Asn	1		
c.242C>T	p.Ser81Leu	2	1	1
c.244G>C	p.Gly82Arg		12	
c.245G>A	p.Gly82Glu		16	
c.254C>A	p.Ala85Asp	1		1
c.283G>A	p.Glu95Lys		1	
c.302T>C	p.Leu101Pro		24	
c.322T>C	p.Trp108Arg	1	7	3
c.324G>T	p.Trp108Cys		2	
c.326G>T	p.Gly109Val	2		
c.346G>A	p.Gly116Arg	2	1	2
c.449T>C	p.Leu150Pro			1
c.457T>G	p.Leu153Val	1		
c.466C>G	p.Gly156Arg			
c.466G>A	p.Gly156Arg	5	11	3
c.481G>A	p.Gly161Ser			4
c.481G>T	p.Gly161Ser		2	
c.482G>A	p.Gly161Asp			1
c.497T>C	p.Leu166Pro	1		
c.518G>A	p.Cys173Tyr	2		
c.533G>A	p.Cys178Tyr	1	6	
c.547G>A	p.Asp183Asn			2
c.560C>T	p.Ser187Phe	1	7	
c.568G>A	p.Gly190Arg	1	20	1
c.583A>C	p.Met195Leu	2		
c.584T>G	p.Met195Arg		26	
c.595G>A	p.Gly199Ser			2
c.598A>G	p.Ile200Val		1	
c.603C>A	p.Asp201Glu		16	6
c.614C>T	p.Ser205Leu	1	6	2
c.628G>A	p.Ala210Pro	1		
c.646G>A	p.Gly216Arg			1
c.653C>T	p.Ser218Leu		4	
c.658A>T	p.Ile220Phe		1	
c.661T>C	p.Ser221Pro		2	
c.697C>T	p.Arg233Cys	2	2	1
c.698G>A	p.Arg233His		2	
c.731T>C	p.Ile244Thr	5	135	8
c.822G>C	p.Glu274Asp		4	
c.823A>C	p.Ser275Arg	1		
c.836T>C	p.Ile279Thr	1	1	
c.846G>C	p.Gln282His	1		2
c.976G>A	p.Val326Ile		2	
c.1020A>G	p.Ile340Met		2	
c.1045G>A	p.Gly349Ser			1
c.1049G>A	p.Gly350Asp		38	
c.1078C>T	p.Arg360Trp		1	
c.1079G>A	p.Arg360Gln		7	3
c.1084G>A	p.Gly362Ser	2	1	
c.1102G>A	p.Ala368Thr		2	
c.1120G>C	p.Asp374His	1		
c.1151T>C	p.Leu384Pro	1	3	

Table S3 Baseline characteristics of PH1 patients and available urinary oxalate ratio (mmol/mmol creatinine)

Median (IQR) or n (%)	No kidney failure at follow-up (n = 71)	Kidney failure at follow-up (n = 13)	P-value	Missing (%)
Age at baseline measurement, years	8.2 [3.4, 18.3]	9.3 [6.6, 15.8]	0.549	0.0
Male sex	45 (63.4)	7 (53.8)	0.734	0.0
Genotype			0.008	2.4
B6 | B6	22 (31.4)	0		
B6 | missense	3 (4.3)	0		
Missense|missense	15 (21.4)	4 (33.3)		
B6 | null	10 (14.3)	2 (16.7)		
Missense | null	0	1 (8.3)		
Null | null	19 (27.1)	3 (25.0)		
Age at first symptoms, years	3.8 [1.5, 6.8]	5.9 [4.0, 6.8]	0.139	15.5
Diagnosis prior to 2010	50 (72.5)	11 (84.6)	0.566	2.4
Age at diagnosis, years	6.0 [3.1, 8.9]	7.3 [5.5, 9.3]	0.298	2.4
Age at follow-up, years	17.7 [9.9, 31.3]	31.1 [11.7, 35.4]	0.419	1.2

Table S4 Baseline characteristics of patients with PH1 and available 24-hour urinary oxalate excretion

Median (IQR) or n (%)	No kidney failure at follow-up (n = 104)	Kidney failure at follow-up (n = 13)	P-value	Missing (%)
Age at baseline measurement, years	10.2 [4.8, 18.6]	12.9 [7.2, 16.8]	0.362	0
Male sex	65 (62.5)	6 (46.2)	0.403	0
Genotype			0.075	1.7
B6 | B6	29 (28.2)	1 (8.3)		
B6 | missense	6 (5.8)	0		
Missense|missense	15 (14.6)	3 (25.0)		
B6 | null	19 (18.4)	0		
Missense | null	5 (4.9)	2 (16.7)		
Null | null	25 (24.3)	4 (33.3)		
Age at first symptoms, years	3.4 [1.5, 6.7]	5.9 [4.8, 8.3]	0.063	41.9
Diagnosis prior to 2010	77 (76.2)	9 (75.0)	1	3.4
Age at diagnosis, years	6.1 [3.4, 11.3]	7.6 [5.8, 9.9]	0.278	3.4
Age at follow-up, years	17.1 [10.6, 24.0]	18.7 [11.7, 37.1]	0.325	1.7

Table S5 Baseline characteristics of PH1 patients with available urinary glycolate ratio

Median (IQR) or n (%)	No kidney failure at follow-up (n = 51)	Kidney failure at follow-up (n = 11)	P-value	Missing (%)
Age at baseline measurement, years (Ugly_R)	8.6 [3.9 – 19.1]	9.3 [6.1 – 16.6]	0.754	0
Male sex	29 (56.9)	6 (54.5)	1.000	0
Genotype			0.036	3.2
B6 | B6	19 (38.0)	0		
B6 | missense	1 (2.0)	0		
Missense|missense	8 (16.0)	4 (40.0)		
B6 | null	7 (14.0)	1 (10.0)		
Missense | null	0	0		
Null | null	14 (28.0)	3 (30.0)		
Age at first symptoms, years	4.3 [1.8 – 7.2]	5.0 [3.9 – 7.1]	0.465	12.9
Diagnosis prior to 2010	43 (86.0)	10 (90.9)	1.000	1.6
Age at diagnosis, years	6.3 [3.3 – 11.3]	7.3 [5.5 – 8.9]	0.844	1.6
Age at follow-up, years	20.3 [12.9– 33.7]	31.1 [12.5-34.0]	0.897	0

Table S6 Baseline characteristics of PH1 patients with available 24-hour urinary glycolate excretion

Median (IQR) or n (%)	No kidney failure at follow-up (n = 75)	Kidney failure at follow-up (n = 12)	P-value	Missing (%)
Age at baseline measurement, years (Ugly_24h)	11.2 [5.7 – 17.5]	10.6 [7.7 – 20.2]	0.662	0
Male sex	42 (56.0)	4 (33.3)	0.251	0
Genotype			0.301	1.1
B6 | B6	21 (28.4)	2 (16.7)		
B6 | missense	4 (5.4)	1 (8.3)		
Missense|missense	10 (13.5)	2 (16.7)		
B6 | null	14 (18.9)	0		
Missense | null	4 (5.4)	2 (16.7)		
Null | null	20 (27.0)	4 (33.3)		
Age at first symptoms, years	4.1 [1.7 – 6.7]	6.3 [4.8 – 8.8]	0.204	51.7
Diagnosis prior to 2010	55 (74.3)	8 (72.7)	1.000	2.3
Age at diagnosis, years	6.2 [3.3 – 11.5]	7.9 [5.9 – 10.4]	0.556	2.3
Age at follow-up, years	16.3 [11.0– 23.6]	21.2 [11.1– 37.9]	0.356	0

Figure S1 Absolute patient numbers included in the OxalEurope Registry

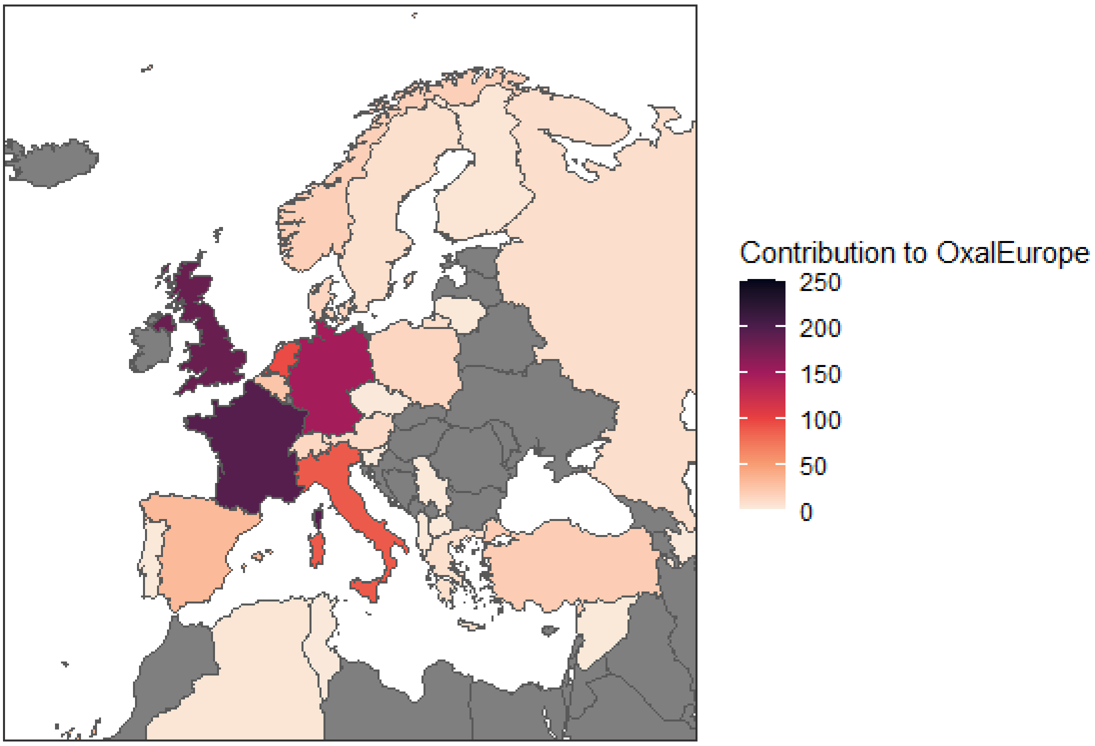

Figure S2 Urinary oxalate for B6+ homozygotes, missense homozygotes and null homozygotes

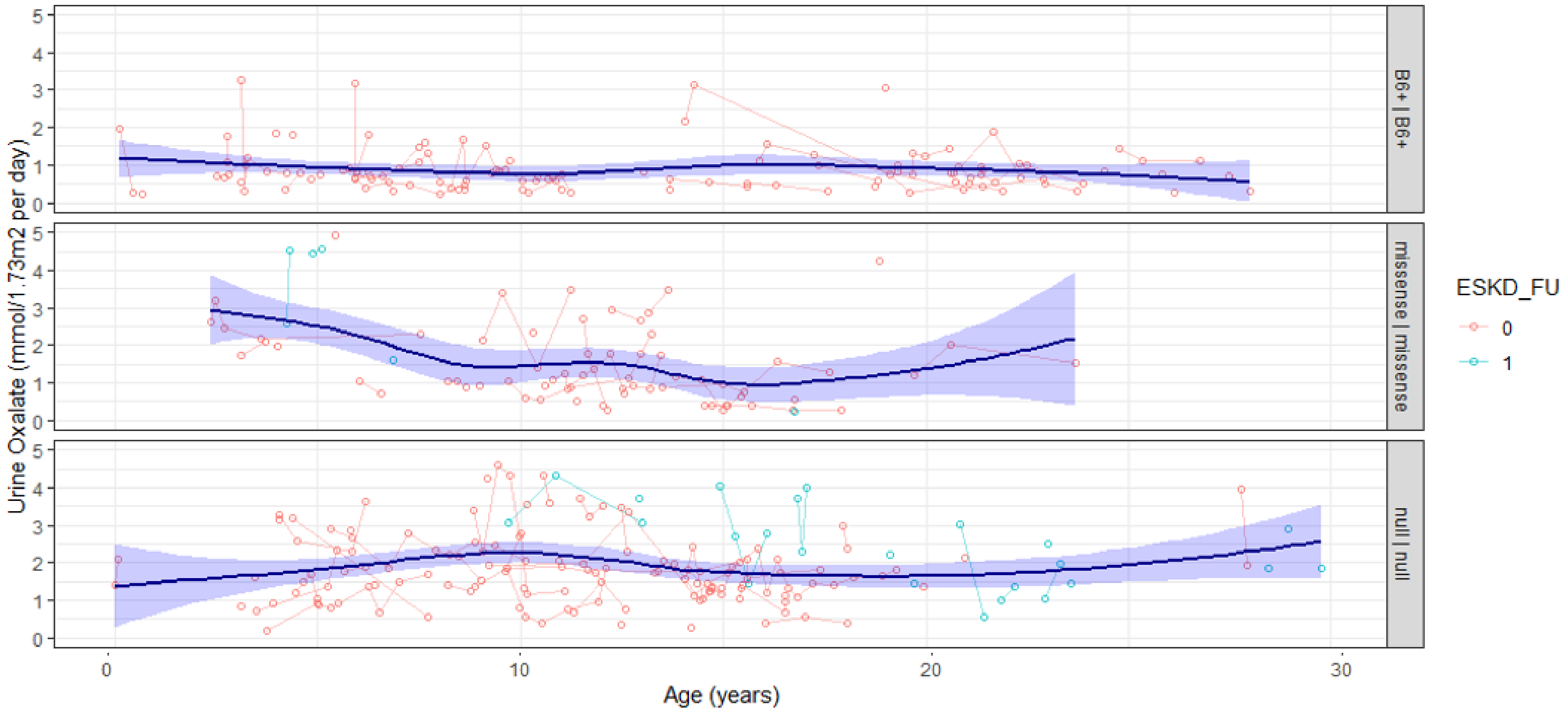

All open circles represent absolute urinary oxalate excretion rates. Green and red circles represent measurements in patients with and without kidney failure at last follow-up (ESKD, end-stage kidney failure = 1 and 0, respectively). Circles connected to each other represent repeated measurements in the same patient. Measurements after onset of kidney failure or after transplantation were excluded.

Figure S3 Urinary glycolate for B6+ homozygotes, missense homozygotes and null homozygotes

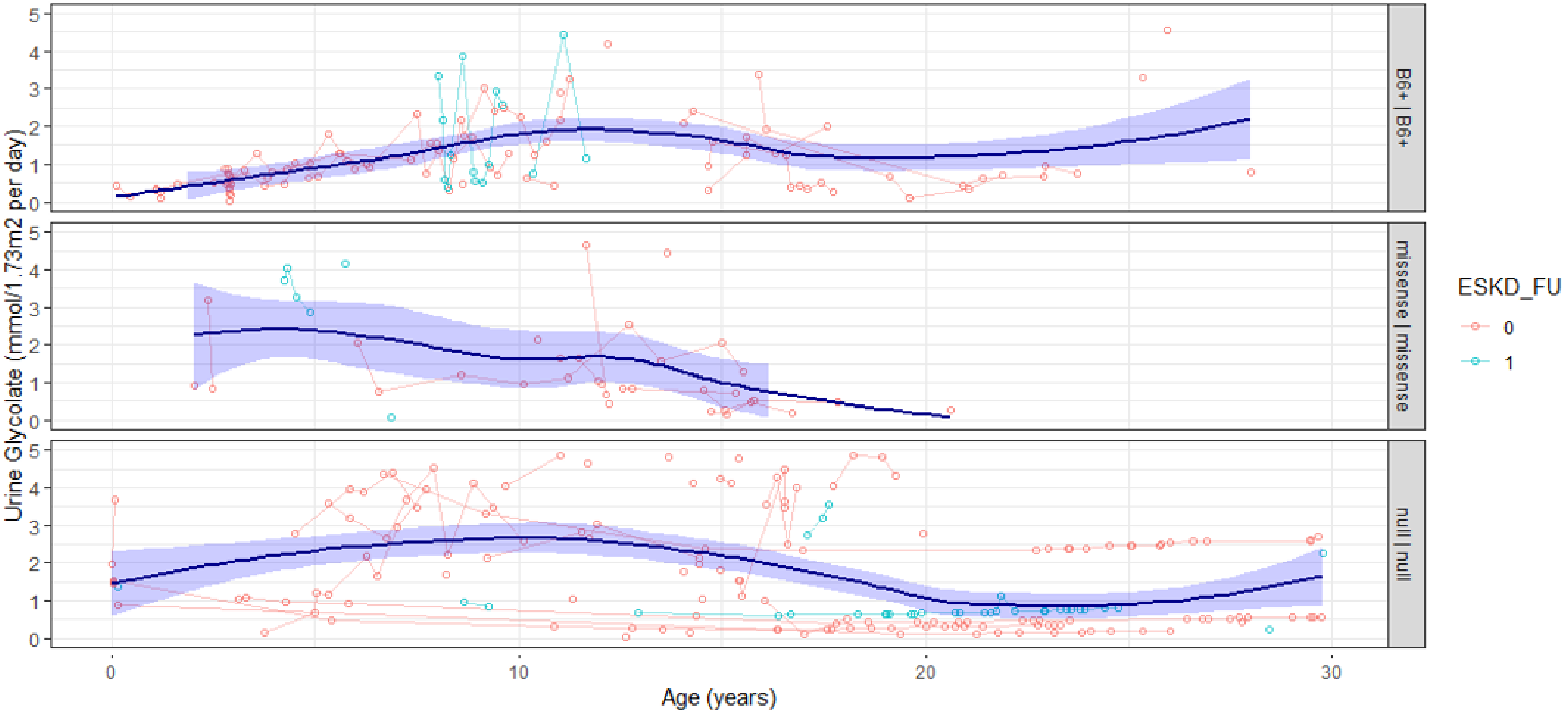

All open circles represent absolute urinary glycolate excretion rates. Green and red circles represent measurements in patients with and without kidney failure at last follow-up (ESKD, end-stage kidney failure = 1 and 0, respectively). Circles connected to each other represent repeated measurements in the same patient. Measurements after onset of kidney failure or after transplantation were excluded.

Reporting checklist for cohort study.

Based on the STROBE cohort guidelines.

Instructions to authors

Complete this checklist by entering the page numbers from your manuscript where readers will find each of the items listed below.

Your article may not currently address all the items on the checklist. Please modify your text to include the missing information. If you are certain that an item does not apply, please write "n/a" and provide a short explanation.

Upload your completed checklist as an extra file when you submit to a journal.

In your methods section, say that you used the STROBE cohortreporting guidelines, and cite them as:

von Elm E, Altman DG, Egger M, Pocock SJ, Gotzsche PC, Vandenbroucke JP. The Strengthening the Reporting of Observational Studies in Epidemiology (STROBE) Statement: guidelines for reporting observational studies.

		Reporting Item	Page Number
**Title and abstract**			
Title	#1a	Indicate the study’s design with a commonly used term in the title or the abstract **p.2**	
Abstract	#1b	Provide in the abstract an informative and balanced summary of what was done and what was found **p.2**	
**Introduction**			
Background / rationale	#2	Explain the scientific background and rationale for the investigation being reported **p.3**	
Objectives	#3	State specific objectives, including any prespecified hypotheses **p.3**	
**Methods**			
Study design	#4	Present key elements of study design early in the paper **p.4**	
Setting	#5	Describe the setting, locations, and relevant dates, including periods of recruitment, exposure, follow-up, and data collection **p.4**	
Eligibility criteria	#6a	Give the eligibility criteria, and the sources and methods of selection of participants. Describe methods of follow-up. **p.4**	

Eligibility criteria	#6b	For matched studies, give matching criteria and number of exposed and unexposed **p.4**
Variables	#7	Clearly define all outcomes, exposures, predictors, potential confounders, and effect modifiers. Give diagnostic criteria, if applicable **p.4**
Data sources / measurement	#8	For each variable of interest give sources of data and details of methods of assessment (measurement). Describe comparability of assessment methods if there is more than one group. Give information separately for for exposed and unexposed groups if applicable. **p.4**
Bias	#9	Describe any efforts to address potential sources of bias **p.4**
Study size	#10	Explain how the study size was arrived at **N/A**
Quantitative variables	#11	Explain how quantitative variables were handled in the analyses. If applicable, describe which groupings were chosen, and why **p.4**
Statistical methods	#12a	Describe all statistical methods, including those used to control for confounding **p.4**
Statistical methods	#12b	Describe any methods used to examine subgroups and interactions **p.4**
Statistical methods	#12c	Explain how missing data were addressed **p.4**
Statistical methods	#12d	If applicable, explain how loss to follow-up was addressed **p.4**
Statistical methods	#12e	Describe any sensitivity analyses **p.4**

Results

Participants	#13a	Report numbers of individuals at each stage of study—eg numbers potentially eligible, examined for eligibility, confirmed eligible, included in the study, completing follow-up, and analysed. Give information separately for for exposed and unexposed groups if applicable. **p.5**
Participants	#13b	Give reasons for non-participation at each stage **N/A**
Participants	#13c	Consider use of a flow diagram **Figure S2**
Descriptive data	#14a	Give characteristics of study participants (eg demographic, clinical, social) and information on exposures and potential

		confounders. Give information separately for exposed and unexposed groups if applicable. **p.5**
Descriptive data	#14b	Indicate number of participants with missing data for each variable of interest **Table 1**
Descriptive data	#14c	Summarise follow-up time (eg, average and total amount) **Table 1**
Outcome data	#15	Report numbers of outcome events or summary measures over time. Give information separately for exposed and unexposed groups if applicable. **p.5**
Main results	#16a	Give unadjusted estimates and, if applicable, confounder-adjusted estimates and their precision (eg, 95% confidence interval). Make clear which confounders were adjusted for and why they were included **p.5-7**
Main results	#16b	Report category boundaries when continuous variables were categorized **p. 5-7**
Main results	#16c	If relevant, consider translating estimates of relative risk into absolute risk for a meaningful time period **N/A**
Other analyses	#17	Report other analyses done—eg analyses of subgroups and interactions, and sensitivity analyses **p.5-7**
**Discussion**		
Key results	#18	Summarise key results with reference to study objectives **p.10**
Limitations	#19	Discuss limitations of the study, taking into account sources of potential bias or imprecision. Discuss both direction and magnitude of any potential bias. **p.12**
Interpretation	#20	Give a cautious overall interpretation considering objectives, limitations, multiplicity of analyses, results from similar studies, and other relevant evidence. **p.10-11**
Generalisability	#21	Discuss the generalisability (external validity) of the study results **p.12**
**Other information**		

Funding	#22	Give the source of funding and the role of the funders for the present study and, if applicable, for the original study on which the present article is based. **p.14**

The STROBE checklist is distributed under the terms of the Creative Commons Attribution License CC-BY. This checklist can be completed online using https://www.goodreports.org/, a tool made by the EQUATOR Network in collaboration with Penelope.ai
